# Experimental investigations on the growth of wall-attached bubble in total dissolved gas supersaturated water

**DOI:** 10.1038/s41598-022-20291-8

**Published:** 2022-09-26

**Authors:** Lu Lin, Ran Li, Jingjie Feng, Shengyun Liu, Qin Zou, Xiaolong Cheng, Honghui Lin

**Affiliations:** 1 State Key Laboratory of Hydraulics and Mountain River Engineering, Sichuan University, Chengdu, Sichuan China; 2 College of Architecture and Civil Engineering, Chengdu University, Chengdu, Sichuan China; 3 POWERCHINA Chengdu Engineering Corporation Limited, Chengdu, Sichuan China

**Keywords:** Environmental sciences, Hydrology, Engineering

## Abstract

Due to dam discharge, waterfalls, sudden increases in water temperature and oxygen production by photosynthesis, the total dissolved gas (TDG) in water is often supersaturated, which may have serious effects on aquatic ecology. When the atmospheric pressure is lower than the TDG pressure in water, the supersaturated dissolved gas in water will slowly release into air. Wall-attached bubbles were formed during the TDG release process. The generation and departure of wall-attached bubbles influence the release process of TDG in water. To simulate the growth period of the wall-attached bubbles under different pressures, a decompression experimental device was designed to record the supersaturated TDG release process. Based on experimental data and mathematical calculations, the quantitative relationship between the bubble growth rate and environmental pressure was obtained. The supersaturated TDG dissipation rate increases monotonically with increasing relative vacuum degree. Applied the calculation method about the wall-attached bubble growth rate, a formula of the supersaturated TDG adsorption flux was proposed, and a prediction method of the TDG release coefficient was established. The simulation results show that with the increasing relative vacuum degree, the TDG release coefficient increases correspondingly, and the adsorption from wall surface area can be obviously promoted. This study provides an important theoretical basis for the accurate calculation of the TDG release process and provides a scientific basis for the accurate prediction of the spatial and temporal distribution of supersaturated TDG under different pressure and solid wall conditions.

## Introduction

In the natural environment, dam discharge, waterfall, sudden rise of water temperature and oxygen production by photosynthesis may lead to total dissolved gas (TDG) supersaturation in water. With cascade hydropower developing and high dam building, dam discharge is becoming the main type of TDG supersaturation. Due to the pressure difference between water and atmosphere, supersaturated TDG in water will slowly release to air. Supersaturation TDG exists for a long time in water, which may lead to bubble disease or even death of fish and cause serious adverse effects on aquatic ecology^1,2^. The release rate of TDG supersaturation is directly related to the extent of the TDG level in water and the TDG pressure difference between water and air^3^, and the findings of the effect on the air entrainment indicated that the energy dissipation efficiency considerably influenced the TDG level^4^. As the hydropower development is moving to high altitude area such as Qinghai-Tibetan Plateau, the release process of supersaturated TDG is quite different from the lower altitude rivers.

In practical engineering applications, there are many solid walls in rivers, such as vegetations under the water and suspended solids in the water, which can easily adsorb the bubble nuclei. The rougher the wall is, the larger the area it provides, and the release process of supersaturated TDG is accelerated^5^. Niu^6^ observed that the release rate of supersaturated TDG increased with an increasing amount of activated carbon in water. The process of the gas phase fraction separating out from water can promote the formation of bubbles, and the characteristic parameters affecting the bubble shape play a key role in the study of TDG mass transfer. Yuan^7^ proposed a supersaturated TDG dissipation model to describe the function of wall adsorption of TDG, and the equation for the adsorption flux of supersaturated TDG over a unit time was achieved from a macroscopic perspective. Based on experiments focusing on the adsorption effect of solid walls, the quantitative relationship between the adsorption coefficient and contact angle of solid surfaces was obtained^8^. Li^9^ experimented on a superheated superhydrophobic surface to study the formation and escape of single bubbles on the vessel wall, and revised Zuber's^10^ prediction equation of the bubble escape diameter and frequency, and obtained the relationship between gas–liquid interface surface tension, liquid density, and equilibrium bubble diameter. In the observation of carbon dioxide dissolved in water, the interaction among bubbles growing in close proximity and the time evolution of the bubble radius was investigated^11,12^. Studies indicated the growth of gas bubbles in a water solution with a supersaturation level that is generally associated with diffusive mass transfer, and the density of the solution sufficiently changes with the gas concentration^13,14^.

Previous studies have shown that solid walls in water can effectively promote the release of supersaturated TDG, and the factors affecting bubble growth include dissolved gas concentration in gas–liquid mixture systems and pressure. To clarify the quantitative relationship between pressure and bubble growth rate on solid walls, a decompression experiment was designed in this paper to investigate the influence of solid wall media on TDG release under different pressure conditions.

## Experimental study on the effect of pressure on bubble growth

### Measurement instruments and method

**Figure 1:**
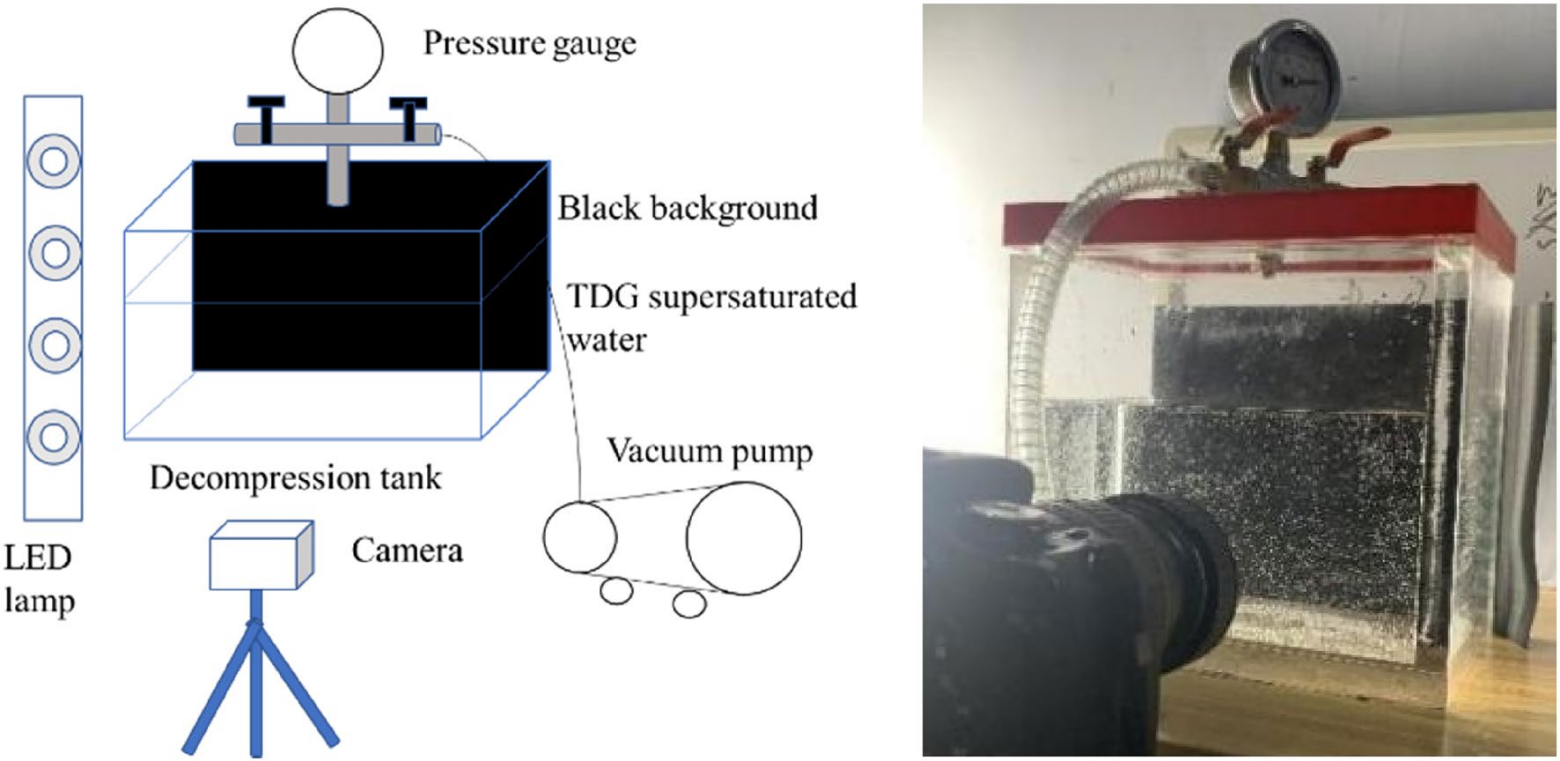
Sketch of the experimental device.

The experiment was conducted in the State Key Laboratory of Hydraulics and Mountain River Engineering of Sichuan University. The experimental device includes rectangular Plexiglas sheet (PMMA) tanks with a length of 220 mm, a width of 160 mm and a height of 210 mm. The air pressure in the water tank was adjusted by a 180 W vacuum pump and a pressure gauge. The water depth in the tank was kept at 120 mm, and the back of the water tank was arranged against a black background to ensure clear wall-attached bubble images during the experiment. The light source was an LED (light emitting diode) lamp with a uniform distribution to the left of the water tank, and the camera was fixed in front of the water tank. A sketch of the experimental device is shown in Fig. 1.

In this experiment, wall-attached bubbles in TDG supersaturated water were observed under vacuum conditions. Bubbles generated in TDG supersaturated water can be adsorbed on the wall and continue to grow up. When the size of wall-attached bubbles reached a certain volume, the bubbles would escape from the wall. According to the observation of the experiment, the growth rate of bubbles can be significantly improved under the condition of reduced pressure. The experiment time for each case was set to be 1 h.

TDG supersaturated water was provided by the supersaturated TDG generation system, which was developed by Sichuan University, China^15^. The system compresses air into tap water^16^, which was used to simulate the river water, and the steady TDG supersaturated water was generated after the air entrained water transport through a multi-U-turn pipe to make the air and water completely mix. The generated TDG supersaturation water was induced into the experimental water tank. The pressure in the experimental water tank was adjusted to a constant vacuum degree, and the temperature was kept at 20 centigrade during the whole experimental process. A digital camera was used to record the precipitation, growth and escape process of wall-attached bubbles. The photographic equipment consisted of a Canon 600D digital camera with 17–85 zoom lens (Taiwan, China), a polarizer and a close-up lens. The TDG in the experimental water tank was measured at the beginning and the end of the experiment with a total dissolved gas pressure (TGP) detector named Pentair Point Four TGP portable trackers (California, U.S.). The TGP measuring range is 0–200%, and the accuracy is 2%. The concentration of TDG dissolved in water can be converted into TDG saturation according to actual needs. The pressure of experiment tank was measured by the pressure gauge set at the top the experimental water tank, with the accuracy of 1.6%.

### Experimental conditions

To express the pressure condition clearly, the relative vacuum degree $\emptyset$ is introduced as Eq. (1).

1 $$\emptyset=1-P/{P}_{0}$$

where $\emptyset$ is the relative vacuum degree, $P$ is the experimental pressure (kPa), and ${P}_{0}$ denotes the local atmospheric pressure (kPa).

The conversion of TDG saturation to TDG concentration is shown in the following equation.

2 $$C={C}^{*}G$$

where $C$ is the TDG concentration (mg L^−1^), ${C}^{*}$ is the equilibrium TDG concentration (mg L^−1^), and $G$ is the saturation of TDG (%).

There were 30 combined working conditions in the experiment. The pressure conditions in this experiment represent the pressure conditions with relative vacuum degrees of 0, 0.2, 0.4, 0.6 and 0.8. For each group of pressure conditions, six TDG initial saturation conditions were set as 110%, 120%, 130%, 140%, 150% and 160%. The water temperature in the experimental tanks was controlled at 20 °C during the whole experimental process. Measurements confirmed that the local atmospheric pressure is 95.5 kPa.

## Results

### Experimental results of TDG concentration

The experimental results of TDG concentration were as Table 1 shows.

**Table 1:** The experimental results of TDG concentration.

Experiment case number	Initial TDG level (%)	Relative vacuum degree $\boldsymbol{\emptyset}$	Initial TDG concentration (mg L^−1^)	TDG concentration after 1 h (mg L^−1^)
1-a	110	0	26.35	26.07
2-a	110	0.2	28.74	27.84
3-a	110	0.4	31.14	29.22
4-a	110	0.6	33.53	32.27
5-a	110	0.8	35.93	34.37
1-b	120	0	38.33	36.53
2-b	120	0.2	26.35	25.33
3-b	120	0.4	28.74	26.87
4-b	120	0.6	31.14	28.32
5-b	120	0.8	33.53	31.28
1-c	130	0	35.93	33.17
2-c	130	0.2	38.33	35.29
3-c	130	0.4	26.35	23.66
4-c	130	0.6	28.74	24.98
5-c	130	0.8	31.14	26.49
1-d	140	0	33.53	28.99
2-d	140	0.2	35.93	30.73
3-d	140	0.4	38.33	33.22
4-d	140	0.6	26.35	21.68
5-d	140	0.8	28.74	22.73
1-e	150	0	31.14	24.08
2-e	150	0.2	33.53	26.18
3-e	150	0.4	35.93	28.20
4-e	150	0.6	38.33	30.07
5-e	150	0.8	26.35	18.93
1-f	160	0	28.74	20.23
2-f	160	0.2	31.14	20.94
3-f	160	0.4	33.53	23.02
4-f	160	0.6	35.93	24.98
5-f	160	0.8	38.33	26.81

The variation in the TDG solubility at different relative vacuum degrees was expressed as:

3 $${C}_{\emptyset}^{*}={C}_{{\emptyset}=0}^{*}(1-\emptyset)$$

where ${C}_{\emptyset=0}^{*}$ represents the TDG solubility at 1 atm (mg L^−1^) and ${C}_{\emptyset}^{*}$ represents the equilibrium TDG concentration at the relative vacuum degree $\emptyset$ (mg L^−1^).

US Army Corps of Engineering^17^ proposed that the dissipation process of supersaturated TDG involves a first-order kinetic reaction. The first-order kinetic reaction is shown as follows:

4 $$\frac{dC}{dt}=-k(C-{C}^{*})$$

where *t* represents the dissipation time (min), $C$ represents the TDG solubility (mg L^−1^); ${C}^{*}$ represents the TDG equilibrium solubility (mg L^−1^) and *k* represents the release coefficient of supersaturated TDG (min^−1^).

**Figure 2:**
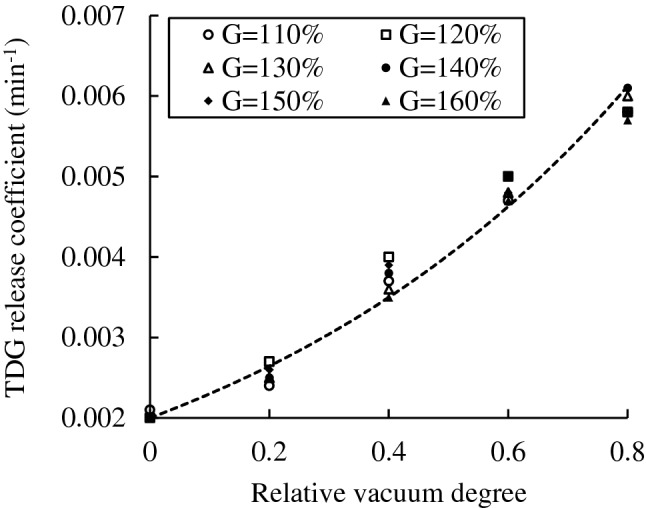
Relationship between the TDG release coefficient and relative vacuum degree.

The release coefficients under different experimental cases were estimated according to the first-order kinetic equation. Figure 2 shows that the release coefficients under different pressure conditions were linearly enhanced with increasing relative vacuum degree.

According to the experimental results under various conditions, the relationship between the TDG release coefficient and the relative vacuum degree by the fitting method is obtained as follows:

5 $${k}_{\emptyset}={k}_{\emptyset=0}{e}^{1.3987\emptyset}$$

where ${k}_{\emptyset}$ represents the TDG release coefficient under relative vacuum degree $\emptyset$ (min^−1^) and ${k}_{\emptyset=0}$ represents the TDG release coefficient under relative vacuum degree 0 (min^−1^).

The coefficient of determination, ${R}^{2}$ is 0.978.

### Image source of wall-attached bubbles

**Figure 3:**
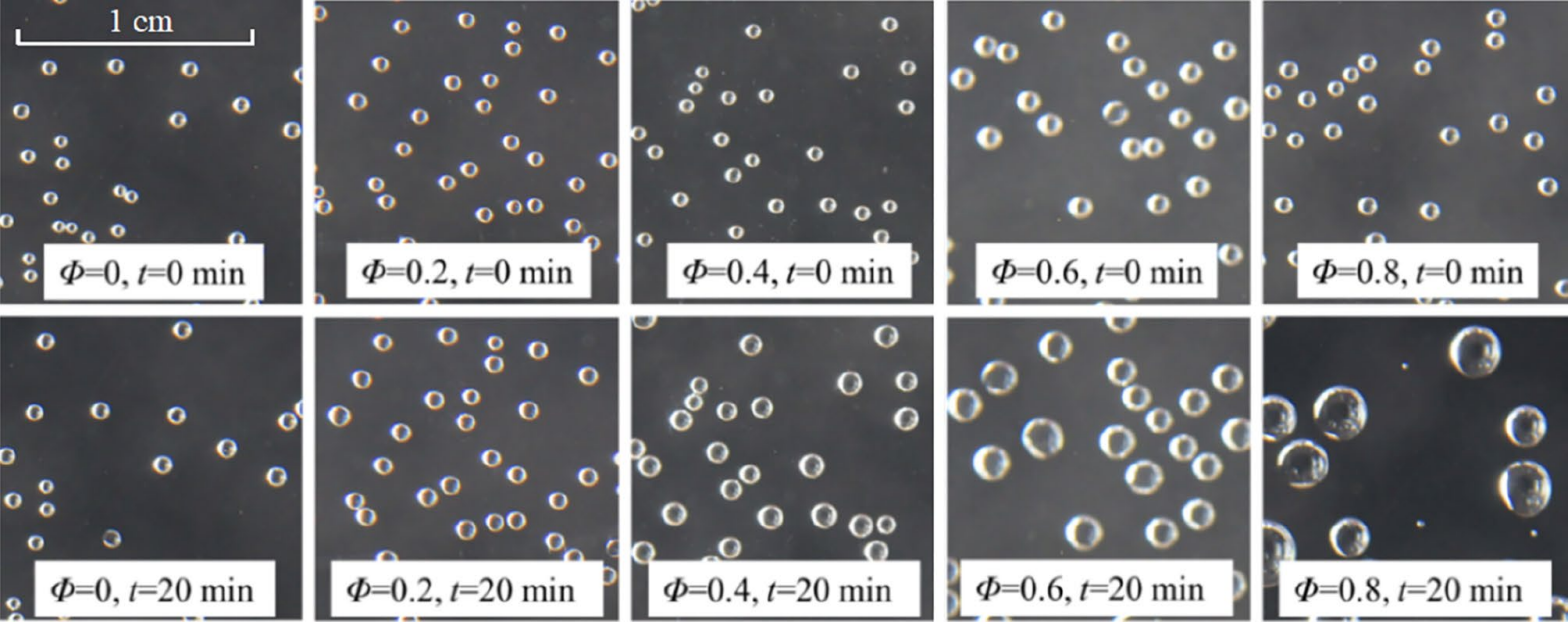
Bubble images of different pressure cases.

The wall-attached bubble images under different pressure cases were taken for each group of experiments. Taking the group with an initial concentration of TDG of 160% as an example, the wall-attached bubble images at different pressure cases were recorded by the camera, as shown in Fig. 3. It was clear that the relative vacuum degree has an obvious promotional effect on the wall-attached bubble growth rate.

By using the image processing method, the wall-attached bubbles in each image were numbered in MATLAB^18^. Image Binarization proposed by Otsu^19^ was used to process the image. The equivalent diameter of bubbles was calculated as follows:

6 $$D=\sqrt{\frac{4{a}_{\mathrm{B}}}{\pi }}$$

where $D$ represents the equivalent diameter of bubbles (mm) and ${a}_{\mathrm{B}}$ represents the projected bubble area (mm^2^).

**Figure 4:**
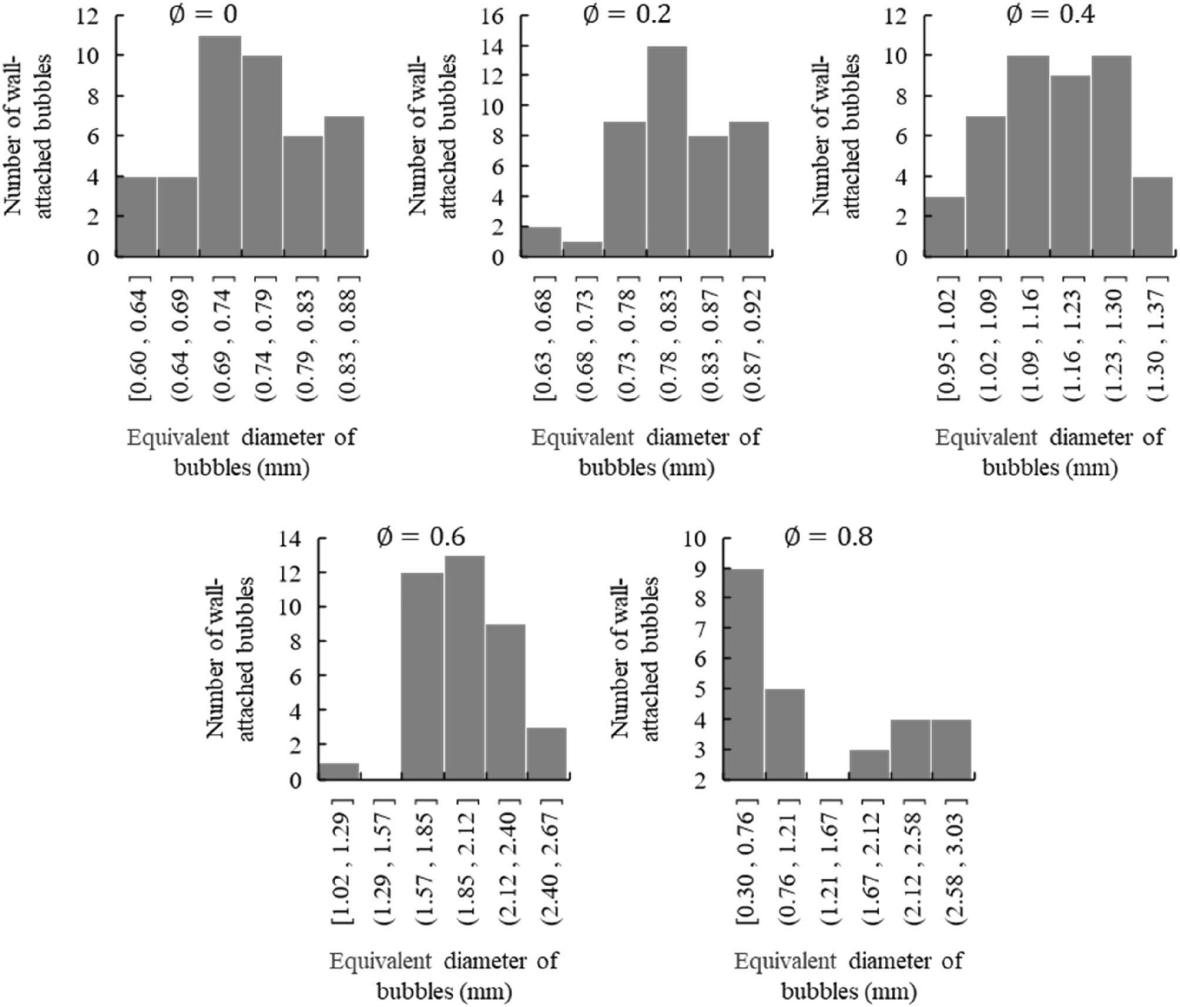
Statistics of bubbles’ equivalent diameter distribution.

The statistics of the bubbles’ equivalent diameter distribution under different pressure conditions are shown in Fig. 4.

According to the statistics of the equivalent diameter of wall-attached bubbles in Fig. 4, the equivalent diameter of wall-attached bubbles has a centralized value at each case. But in the pressure group 5 ($\emptyset=0.8$), as the wall-attached bubbles grown faster than the other pressure groups, they experienced departure and regrowth withing 20 min, which cause the equivalent diameter of the wall-attached bubbles exhibits a double peak distribution.

## Discussion

### The role of wall-attached bubbles in the supersaturated TDG release coefficient

**Figure 5:**
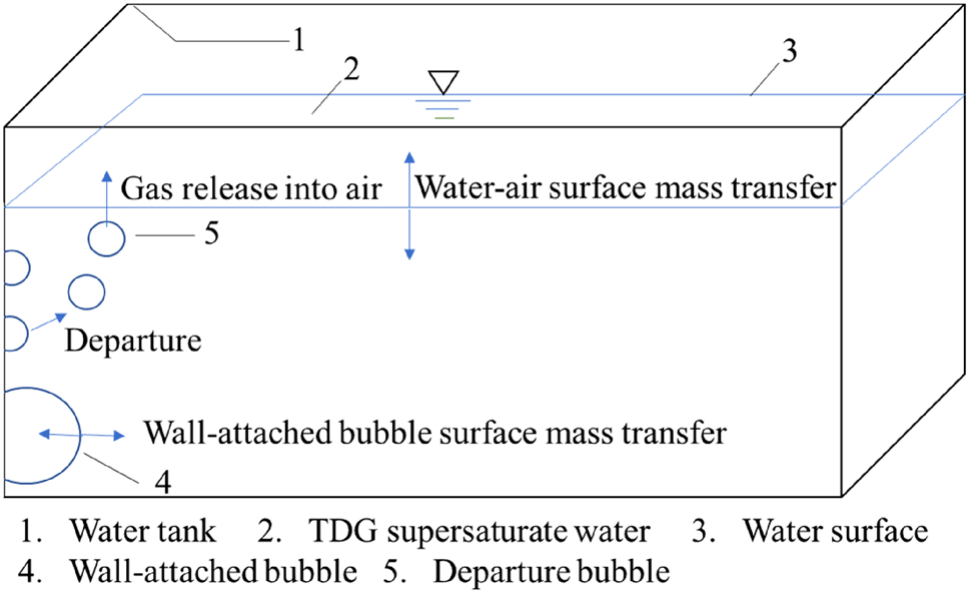
Sketch of the TDG mass transfer.

Based on a previous study on the wall adsorption effect on the TDG release process, the calculation method of the supersaturated TDG release coefficient based on wall-attached bubbles can be used to predict the TDG release process. The release process of supersaturated TDG in water consists of three parts^7^. However, in static water, there are almost no free bubbles in water, so the TDG release process can be simplified into two parts: air–water mass transfer and wall adsorption, as shown in Fig. 5.

The amount of supersaturated TDG released in static water can be expressed as:

7 $${F}_{\mathrm{G}}={F}_{\mathrm{s}}+{F}_{\mathrm{w}}$$

where ${F}_{\mathrm{G}}$ represents the release rate of supersaturated TDG, (mg L^−1^ min^−1^); ${F}_{\mathrm{s}}$ represents the release rate of supersaturated TDG from air–water mass transfer, (mg L^−1^ min^−1^); and ${F}_{\mathrm{w}}$ represents the release rate of supersaturated TDG from wall adsorption, (mg L^−1^ min^−1^).

1. The release rate of supersaturated TDG from air–water mass transfer is:
  8 $${F}_{\mathrm{G}}=k(C-{C}_{\emptyset}^{*})$$
  where *k* represents the TDG release coefficient (min^−1^); $C$ represents the concentration of supersaturated TDG (mg L^−1^); and ${C}_{\emptyset}^{*}$ represents the equilibrium concentration of supersaturated TDG (mg L^−1^).
2. The release rate of supersaturated TDG from wall adsorption:
  9 $${F}_{\mathrm{s}}={K}_{\mathrm{s}}{a}_{\mathrm{s}}(C-{C}_{\emptyset}^{*})$$
  where ${K}_{\mathrm{s}}$ represents the mass transfer coefficient of the air–water interface (m·min^−1^) and ${a}_{\mathrm{s}}$ represents the specific surface area of supersaturated water (m^−1^).
  The quantitative relationship between the mass transfer coefficient of the air–water interface and the surface turbulent kinetic energy was proposed by Li^20^.
  10 $${K}_{\mathrm{s}}=0.085{{T}_{\mathrm{s}}}^{1/2}\text{+0.0014}$$
  where ${T}_{\mathrm{s}}$ represents the surface turbulent kinetic energy (m^2^ s^−2^).
3. The formula for calculating the adsorption rate of TDG by solid walls in supersaturated water is as follows:
  11 $${F}_{\mathrm{w}}=\frac{d{M}_{\mathrm{w}}}{dt}{a}_{\mathrm{d}}$$
  where ${a}_{\mathrm{d}}$ represents the specific solid wall area of supersaturated water (m^−1^). ${M}_{\mathrm{w}}$ represents the TDG adsorption flux, (mg m^−2^), which can be expressed as the sum of the amount of TDG contained in the wall-attached bubbles and the amount of TDG released from the escaped bubbles.

The release process of supersaturated TDG in static water includes mass transfer at the water–air interface, internal release of TDG in water and mass transfer at the wall-attached bubble interface. The formula of TDG adsorption flux can be expressed as follows:

12 $${M}_{\mathrm{w}}={M}_{\mathrm{B}}+{M}_{\mathrm{d}}$$

where ${M}_{\mathrm{W}}$ represents the TDG adsorption flux (mg m^−2^), ${M}_{\mathrm{B}}$ represents the amount of TDG contained in the wall-attached bubbles (mg m^−2^) and ${M}_{\mathrm{d}}$ represents the amount of TDG released from the escaped bubbles (mg m^−2^).

The amount of TDG contained in the wall-attached bubbles ${M}_{\mathrm{B}}$ was defined as the wall-attached bubble adsorption flux, and *N* was defined as the wall-attached bubble number density, the calculation formula is as follows:

13 $${M}_{\mathrm{B}}={\rho }_{\mathrm{B}}{V}_{\mathrm{B}}N\times {10}^{-2}$$

where ${\rho }_{\mathrm{B}}$ represents the air density in wall-attached bubbles, (mg L^−1^); ${V}_{\mathrm{B}}$ represents the wall-attached bubble volume, (mm^3^); *N* represents the wall-attached bubble number density, (cell cm^−2^).

The amount of TDG released from the escaped bubbles ${M}_{\mathrm{d}}$ was defined as the bubble escapable adsorption flux, and the calculation formula is as follows:

14 $${M}_{\mathrm{d}}={\rho }_{\mathrm{B}}{V}_{\mathrm{d}}N\times {10}^{-2}$$

where ${V}_{\mathrm{d}}$ represents the wall-attached bubble departure volume, (mm^3^).

The TDG release coefficient under the influence of a solid wall was simplified as follows:

15 $$k=\frac{\frac{d{M}_{\mathrm{w}}}{dt}{a}_{\mathrm{d}}+\left(C-{C}^{*}\right){K}_{\mathrm{s}}{a}_{\mathrm{s}}}{(C-{C}^{*})}$$

where *k* represents the TDG release coefficient, (min^−1^); ${M}_{\mathrm{w}}$ represents the TDG adsorption flux, (mg m^−2^); $t$ represents time, (min); ${a}_{\mathrm{d}}$ represents the specific solid wall area of supersaturated water (m^−1^); $C$ represents the concentration of supersaturated TDG (mg L^−1^); ${C}_{\emptyset}^{*}$ represents the equilibrium concentration of supersaturated TDG (mg L^−1^); ${K}_{\mathrm{s}}$ represents the mass transfer coefficient of the air–water interface (m min^−1^) and ${a}_{\mathrm{s}}$ represents the specific surface area of supersaturated water (m^−1^).

### Analysis of the growth rate of the wall-attached bubble

**Figure 6:**
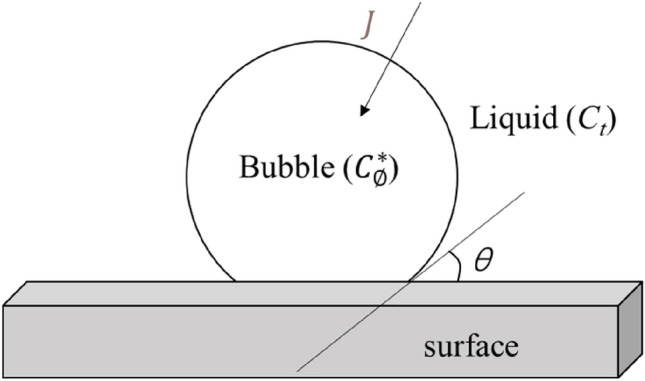
Mass transfer process of TDG on the surface of wall-attached bubbles.

Due to the release process of supersaturated TDG in water, the wall-attached bubble diameter increases gradually. The mass transfer process of TDG on the surface of wall-attached bubbles is shown in Fig. 6.

The air mass transfer flux from the liquid to bubbles can be expressed as follows:

16 $$J=K(C-{C}_{\emptyset}^{*})$$

where *J* represents the air mass transfer flux from the liquid to bubbles (mg mm^−2^ min^−1^); *K* represents the mass transfer coefficient at the bubble surface (mm min^−1^); *C* represents the TDG concentration (mg L^−1^); and ${C}_{\emptyset}^{*}$ represents the equilibrium TDG concentration under relative vacuum degree $\emptyset$ (mg L^−1^).

By substituting Eq. (4) into Eq. (16), the mass transfer flux of supersaturated TDG on the bubble surface can be expressed as:

17 $$J=K\left[\left({C}_{0}-{C}_{\emptyset}^{*}\right){e}^{-kt}\right]$$

where ${C}_{0}$ represents the initial TDG solubility, (mg L^−1^).

According to mass conservation, the relationship between the TDG release process and wall-attached bubble volume growth rate can be expressed as:

18 $$\frac{d{V}_{B}}{dt}=\frac{K{A}_{\mathrm{B}}}{{\rho }_{\mathrm{B}}}\left[\left({C}_{0}-{C}_{\emptyset}^{*}\right){e}^{-kt}\right]$$

where ${V}_{\mathrm{B}}$ represents the wall-attached bubble volume, (mm^3^); ${A}_{\mathrm{B}}$ represents the wall-attached bubble surface area, (mm^2^); and ${\rho }_{B}$ represents the air density in wall-attached bubbles, (mg L^−1^).

By applying the boundary condition that when $t=0$, ${V}_{\mathrm{B}}=0$, the differential Eq. (18) can be solved as Eq. (19).

19 $${V}_{\mathrm{B}}=\frac{K{A}_{\mathrm{B}}\left({C}_{0}-{C}_{\emptyset}^{*}\right)}{{\rho }_{\mathrm{B}}k}\left(1-{e}^{-kt}\right)$$

**Figure 7:**
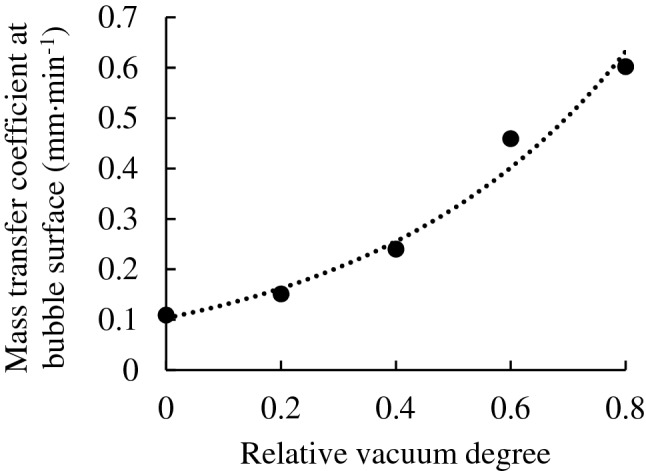
Relationship of the bubble surface mass transfer coefficient and the relative vacuum degree.

Due to the contact angle between water, gas and solids, the shape of wall-attached bubbles is not a complete sphere^21^. The volume of the wall-attached bubbles was calculated accurately from the images accounting for the contact angle effect, as described by Lin^18^. Thereafter, the mass transfer coefficient at bubble surface *K* in Eq. (19) under different relative vacuum degrees $\emptyset$ can be calculated. The relationship between $K$ and $\emptyset$ is shown in Fig. 7.

According to the experimental results under various conditions, the quantitative relationship between *K* and $\emptyset$ by the fitting method was obtained as follows:

20 $${K}_{\emptyset}={K}_{\emptyset=0}{e}^{2.1755\emptyset}$$

where ${K}_{\emptyset}$ represents the mass transfer coefficient at the bubble surface under a relative vacuum degree of $\emptyset$ (mm min^−1^) and ${K}_{\emptyset=0}$ represents the mass transfer coefficient at the bubble surface under a relative vacuum degree of $\emptyset=$ 0 (mm min^−1^).

The coefficient of determination, ${R}^{2}$ is 0.985.

### Analysis of the number density of wall-attached bubbles

**Figure 8:**
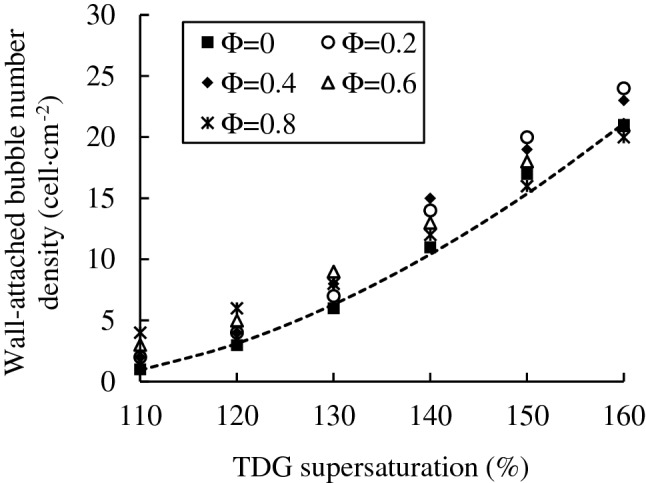
Statistics of the wall-attached bubble number density.

The number density (cell cm^−2^) of wall-attached bubbles for each experimental case can be obtained by program calculation in MATLAB, and the statistical results are shown in Fig. 8.

According to the experimental results, the supersaturation level of TDG is the key factor that influences the wall-attached bubble number density. The quantitative relationship of the wall-attached bubble number density increasing with TDG supersaturation by the fitting method is obtained as follows:

21 $$N=0.017{(G-100)}^{1.74}$$

where *N* represents the wall-attached bubble number density (cell cm^−2^). *G* represents the supersaturation of TDG, (%).

The coefficient of determination, ${R}^{2}$ is 0.997.

### Analysis of wall-attached bubble departure in static water

According to observations during experiments, wall-attached bubbles departure from the wall to the water surface when they grow to a certain diameter^22^.

The volume of the departure bubbles comprises the important part of the release of TDG in water. The departure time of wall-attached bubbles was defined as $X$, which represents the retention time from the formation to the departure. The experiment water tank was static so the mass transfer from nucleated bubbles was considered as diffusion, and the effect of advection was not considered in this study. According to the statistical results regarding the relative vacuum degree of 0.8 in the experiment, the departure diameter of wall-attached bubbles ranged from 1.5 to 2.3 mm, and the average bubble departure diameter in static TDG supersaturated water was 1.94 mm. The statistical results were consistent with Brennen's^23^ conclusion that all bubble nuclei would grow to the same maximum radius. As the value of wall-attached bubble departure diameter was very small, and the appearance of bubble rising was infrequent in the static experiment observation, the entrainment enhancing water flow of rising bubbles was not considered in this study. According to the quantitative relationship between the TDG release process and the wall-attached bubble volume growth rate, the value of $X$ can be calculated as follows.

22 $$X=-\frac{1}{k}\mathrm{exp} \left(1-\frac{{V}_{\mathrm{d}}{\rho }_{\mathrm{B}}k}{K{A}_{\mathrm{B}}\left({C}_{0}-{C}_{\emptyset}^{*}\right)}\right)$$

The departure frequency of wall-attached bubbles was defined as follows:

23 $$f=\frac{N}{X}$$

where $f$ represents the departure frequency of wall-attached bubbles, (min^−1^ cm^−2^) and *N* represents the wall-attached bubble number density, (cell cm^−2^).

**Figure 9:**
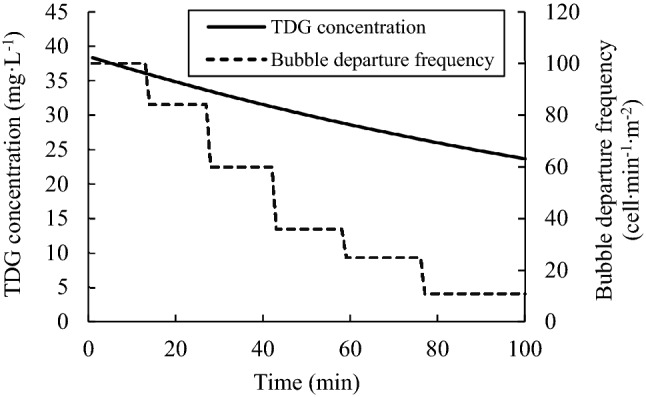
Simulation result of the wall-attached bubbles’ departure frequency.

The departure frequency of wall-attached bubbles can be simulated as shown in Fig. 9. In the simulation results, the departure frequency of wall-attached bubbles decreased obviously with decreasing TDG concentration.

### Calculation of the adsorption flux through the wall-attached bubbles

**Figure 10:**
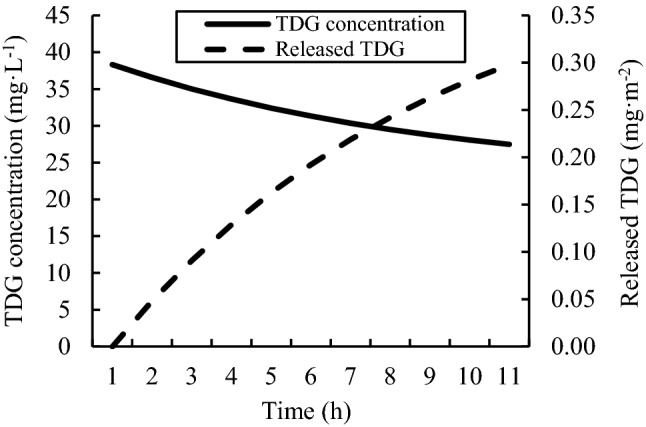
The simulation of TDG adsorption flux ($\emptyset=0$).

Under environmental conditions of a relative vacuum degree $\emptyset=0$, the bubble growth rate was slow, and it was difficult for wall-attached bubbles to escape. By considering the TDG release process, the TDG adsorption flux under this condition can be simulated, as shown in Fig. 10. With TDG concentration decreasing, the growth rate of wall-attached bubbles gradually slowed down, and the wall adsorption rate of supersaturated TDG decreased over time.

**Figure 11:**
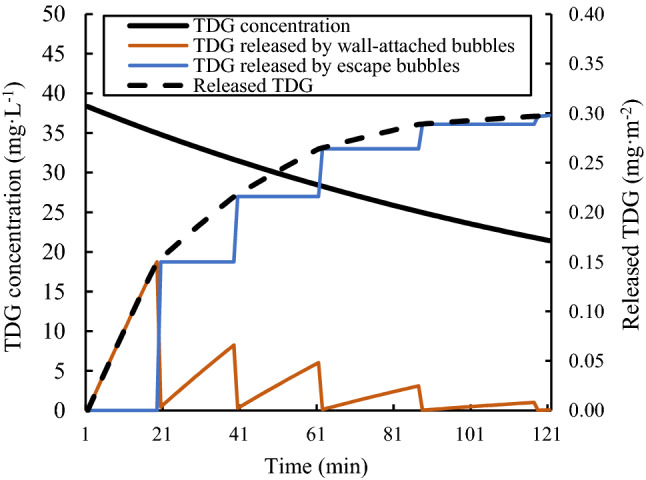
Simulation of the supersaturated TDG adsorption flux ($\emptyset=0.8$).

The growth period was defined to describe the time of a group of wall-attached bubbles from appearance to departure. In the following simulations, the statistical value of a group of wall-attached bubbles’ average departure time was used to represent the value of the growth period. At a high value of the relative vacuum degree ($\emptyset=0.8$), due to the promotion effect of pressure on the bubble growth rate, the growth period of wall-attached bubbles was significantly shortened. Considering the influence of bubble growth and escape on supersaturated TDG release, the total amount of TDG adsorption flux under this condition can be simulated, as shown in Fig. 11.

### The simulation cases of different pressure condition

**Figure 12:**
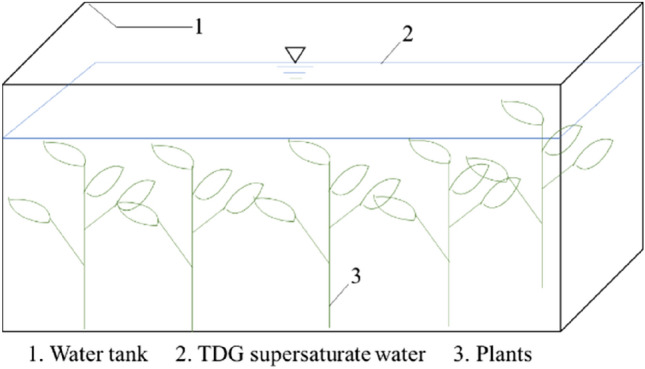
The simulated water tank.

The simulation is based on a water tank that has different amounts of plants, and the air pressure over the water tank depends on the local altitude. The volume of water was assumed to be 1 m^3^, and the initial supersaturated TDG was assumed to be 150% for the modeling calculation. The diagram of the simulated water tank is shown in Fig. 12.

**Figure 13:**
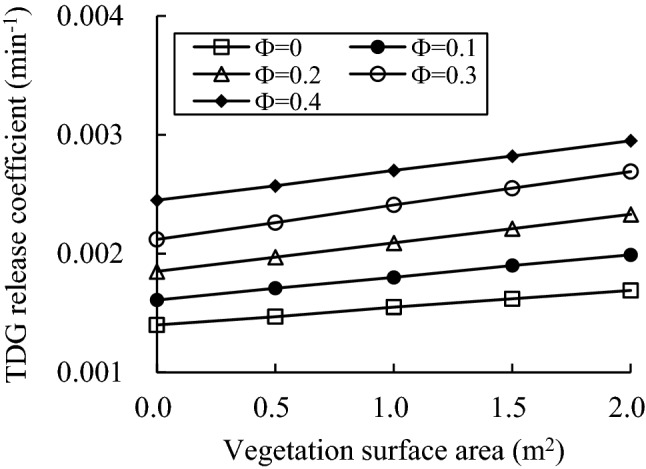
Calculation results of the TDG release coefficient.

The simulation cases of the pressure condition considered 5 cases with relative vacuum degrees of 0, 0.1, 0.2, 0.3 and 0.4. As the calculation result of Eq. (10), the mass transfer coefficient of the air–water interface in the case of static water was 0.0014 min^−1^. According to the leaf surface contact angle of the vegetation in natural water, a contact angle of 90° was chosen for simulation. The departure diameter of wall-attached bubbles can be calculated as 3.01 mm^18^. The initial TDG release coefficient in the case of different pressures was calculated according to Eq. (5), and the simulated TDG release time was 2 h. The TDG release coefficients under different vegetation superficial areas and different pressure conditions were calculated, as shown in Fig. 13.

The simulation results show that both pressure and vegetation surface area can promote the release process of supersaturated TDG. Under lower pressure, the effect of vegetation surface area on the TDG release coefficient can be more obvious.

## Conclusions

In practical engineering applications, there are many solid walls in lakes and rivers, such as vegetation and suspended solids, which easily adsorb bubbles and promote wall-attached bubble growth. An experimental water tank with controllable pressure was designed in this paper to study the release process of supersaturated TDG under different environmental pressures and the function of walls to TDG dissipation. The quantitative relationship between the relative vacuum and the TDG release coefficient was obtained. By making analyses of the wall-attached bubbles growth rate and departure frequency, the quantitative relationship between the wall adsorption flux of TDG and the wall-attached bubbles growth period was obtained. To discuss the effect of solid wall adsorption on the TDG release coefficient, a prediction of the TDG release coefficient accounting for the wall adsorption effect was proposed. Based on the experimental research conclusions, the effects of the solid wall surface and pressure on the TDG release process were simulated, and the results show that with the increasing relative vacuum degree, the TDG release coefficient increases correspondingly, and the adsorption mechanism of solid wall area can be obviously promoted under lower environmental pressure.

The study in this paper provides a new angle of view to research on the TDG release mechanism. The results could provide basic data and scientific theory to evaluate the TDG release process in difference atmosphere and promotion of wall.

## List of symbols

*a*_B_: Projected bubble area (mm^2^)
*a*_d_: Specific solid wall area of supersaturated water (m^−^^1^)
*a*_s_: Specific surface area of supersaturated water (m^−^^1^)
*A*_B_: Wall-attached bubble surface area (mm^2^)
*C**: Equilibrium TDG concentration (mg L^−^^1^)
*C*: TDG concentration (mg L^−^^1^)
*D*: Equivalent diameter of bubbles (mm)
*D*_d_: Wall-attached bubble’s departure volume (mm^3^)
*f*: Departure frequency of wall-attached bubbles (min^−^^1^∙cm^−^^2^)
*F*_G_: Release rate of supersaturated TDG (mg L^−^^1^ min^−^^1^)
*F*_s_: Release rate of supersaturated TDG from air–water mass transfer (mg L^−^^1^ min^−^^1^)
*F*_w_: Release rate of supersaturated TDG from wall adsorption (mg L^−^^1^ min^−^^1^)
_*G*_: Saturation of TDG (%)
*J*: Air mass transfer flux from the liquid to bubbles (mg mm^−2^ min^−1^)
*k*: TDG dissipation coefficient (min^−^^1^)
*K*: Mass transfer coefficient at bubble surface (mm min^−1^)
*K*_s_: Mass transfer coefficient of air–water interface (m min^−^^1^)
*M*_w_: TDG adsorption flux (mg m^−2^)
*M*_B_: Wall-attached bubble adsorption flux (mg m^−2^)
*M*_d_: Departure bubble adsorption flux (mg m^−2^)
*N*: Wall-attached bubble number density (cell cm^−2^)
*P*: Experiment pressure (kPa)
*P*_0_: Standard atmospheric pressure (kPa)
*t*: Time (min)
*Ts*: Surface turbulent kinetic energy (m^2^ s^−2^)
*V*_B_: Wall-attached bubble volume, (mm^3^)
*V*_d_: Departure bubble volume (mm^3^)
*X*: Bubble retention time from the formation to the departure (min)
$\phi$: Relative vacuum degree
${\rho }_{B}$: Air density in wall-attached bubble (mg L^−1^)
